# Intervertebral Foramen – A Gateway to Epidural Space in Severe Lumbar Scoliosis

**DOI:** 10.5152/TJAR.2023.21351

**Published:** 2023-04-01

**Authors:** Sandeep Diwan, Abhijit Nair, Parag Sancheti

**Affiliations:** 1 Department of Anaesthesiology, Sancheti Hospital, Pune, Maharashtra State, India; 2 Department of Anaesthesiology, Ibra Hospital, Ibra-414, Sultanate of Oman; 3 Department of Orthopaedics, Sancheti Hospital, Pune, Maharashtra State, India

**Keywords:** Computed tomography, epidural, regional anaesthesia, scoliosis, surgery

## Abstract

We describe cases in which a preoperative computed tomography was used to guide the placement of an epidural catheter through the defect in the intervertebral foramina in patients with severe lumbar scoliosis. We demonstrate the adroitness with which epidural catheters were inserted through the intervertebral foramina. Computed tomography scan illustrates and plots the needle path creating a 3-dimensional image of the vertebral body rotation, needle trajectory, and the distance from the skin to the intervertebral foramina. Severe scoliosis is defined as a lateral curvature (Cobb’s angle) of more than 50 degrees. It was proposed in severe idiopathic scoliosis that interventional pain management techniques are managed with fluoroscopic imaging or an alternative form. However, after a computed tomography evaluation of the scoliotic spine, we assumed that the intervertebral foraminal anatomy would facilitate a safe and efficient epidural needle and subsequent catheter positioning in severe scoliotic patients.

**Main Points**

- Performing central neuraxial block in patients having scoliosis is challenging.
- Fluoroscopic guidance can be helpful in such patients for a successful central neuraxial block.
- Identifying the intervertebral foramen using computed tomography could aid in assessing the epidural space in patients with scoliosis.

## Introduction

Neuraxial techniques in patients with severe scoliosis are arduous and precarious. Imaging techniques and ultrasound-guided access to neuraxial space are recommended. Case reports and series advocate a paramedian approach in patients with moderate scoliosis.^1,2^ An algorithmic approach is proposed for neuraxial techniques in scoliosis patients. We report our experience with 2 patients who underwent lower-limb surgeries. We used the lumbar intervertebral foraminal approach as a gateway to neuraxial space to insert epidural catheters for perioperative anaesthesia and analgesia. The case highlights the importance of an appropriate plan with a preoperative computed tomography (CT) scan and a thorough understanding of the scoliotic segment of the vertebral body.

## Case Presentation

**Figure 1.**
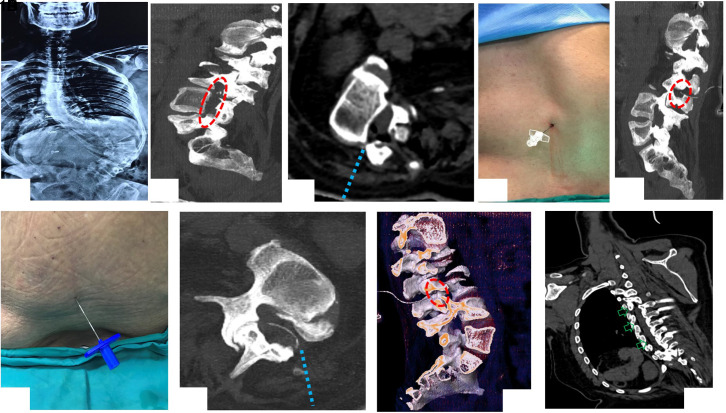
(A) Patient 1—x-ray of lumbar epidural scoliosis, (B) Patient 1—CT scan sagittal section depicting inter-vertebral foraminal defect (red dotted circle-proposed needle Tuohy entry), (C) Patient 1—CT scan axial section demonstrating the proposed needle entry (blue-dotted line) through the intervertebral foramina, (D) Patient 1—Tuohy needle insertion on the left of the spinous process and convex side of the scoliosis, (E) Patient 1—Catheter depicted entering the intervertebral foramina beneath the pedicle (red-dotted circle), (F) Patient 2—Tuohy needle insertion on the right of the spinous process and convex side of the scoliosis, (G) Patient 2—CT scan axial section demonstrating the proposed needle entry (blue-dotted line) through the inter-vertebral foramina and with the catheter entering from inter-vertebral foramina into the epidural space, (H) Patient 2—A volume rendering technique of CT scan depicting catheter entering the intervertebral foramina beneath the pedicle (red-dotted circle), (I) Patient 2—Hypoechoic linear distribution (air) along the convex side of the scoliosis demonstrating the epidural path (green arrows).

In 2 patients, a continuous lumbar epidural (CLE) was planned for surgical procedures. One of the patients (patient 1) refused a peripheral nerve block for fear of multiple needle pricks, and the surgeon wanted to evaluate motor functions in the immediate post-operative period. Patient 2 suffered from long-standing interstitial fibrosis with room air saturation of 89% and was oxygen dependent, bearing in mind post-operative pain relief a CLE was chosen. Thoracolumbar x-rays depicted scoliosis (Cobb’s angle more than 50) in both patients. [Figure 1A (patient 1)]. Following a discussion with a radiologist, after obtaining informed consent, both patients were counseled for pre-operative and a possible post-operative CT scan. Computed tomography scans of the lumbar spine performed were reviewed for possible access to the neuraxial space.

### Patient 1

Discussion with the radiologist concluded that the defects in the intervertebral foramina at the level of L3-4 in patient 1 (Figure 1B) can be approached from lateral to the spinous process at a distance of 3-4 cm. The needle trajectory was calculated, and the depth of the intervertebral foramina measured in CT axial images was 4 cm, and the target area was the posterior and lateral aspect of the intervertebral foraminal defect (Figure 1C). In a sitting position after skin infiltration with 1% lidocaine, an 18 g Tuohy needle was inserted, 4 cm lateral to the spinous process on the right side at the level of L3-4 (Figure 1D), in a trajectory as determined on the CT scan image (axial plane). At a distance of 4-4.5 cm from the skin, a click was appreciated. Loss of resistance to air identified the epidural space.

### Patient 2

In a sitting position after skin infiltration with 1% lignocaine, an 18 g Tuohy needle was inserted 5 cm lateral to the spinous process on the left side (Figure 1F). As was planned from the CT scan axial image, the defect in the intervertebral foramina on the right convex side of scoliosis was targeted at the level of L2-3 (Figures 1G and 1H). The needle trajectory was ascertained and the depth of the intervertebral foramina measured in CT axial images was 5.5 cm (Figure 1G), and the target area was the posterior and lateral aspect of the intervertebral foraminal defect (Figure 1G). From skin at a distance of 5.5 cm, a pop was recognised suggesting the tip of the needle transgressed the intra-foraminal ligaments. A loss of resistance to air established the needle tip in the epidural space.

In both patients, following a test dose (3 mL of 1.5% lidocaine with 1:200 000 adrenaline), epidural catheter was advanced 5 cm past the tip of the needle and secured with a sterile dressing to the skin. A second test dose injected through the catheter was negative for intravascular and intrathecal injection. In both the patients, after an initial dose of 0.5% bupivacaine, 10 mL injection through the catheter was given. In patient 1, since the analgesic effect was more on the non-surgical side, a top-up of 7mL 0.5% bupivacaine was injected. Block performance time (local anaesthetic injection to patient supine) was 22 minutes in patient 1 and 9 minutes in patient 2. Surgical procedures were uncomplicated.

Post-operative a CT contrast was performed with omnipaque contrast dye (0.2 mL-dead space of epidural catheter), which revealed a catheter entering the epidural space through the intervertebral foraminal defect (1E) at the level of L3-4 in patient 1 and beneath the pedicle in patient 2 (Figure 1G) at the level of L2-3. In patient 2, a linear hypoechoic distribution of air was observed along the convex side of scoliosis, which is the unilateral epidural pathway. The spinal cord tissue was on the concave side.

Continuous infusion of 0.1% ropivacaine at 6 mL hr^-1^ was administered in both the patients supplemented with 1 g intravenous paracetamol every 8 hours, providing excellent pain control with a VAS of not more than 4 until the catheters were removed at 48 hours.

## Discussion

Two concerns haunt neuraxial techniques in scoliotic patients. First is the needle tip placement in the neuraxial space, and the second is the LA distribution. With a proposed mid-line approach^1^ and needle angled towards the convex side of the curve, increased volume of LA was required for a satisfactory block, as contrast flow distributed along the convexity (19%). Though the interspinous approach is most favoured for neuraxial anaesthesia, in severe lumbar scoliosis (Cobb’s angle > 50), due to the almost horizontal placement of the spinous process concerning the skin, an alternative to the interspinous approach would be necessary. Moreover, studies have shown spinous process is not a reliable marker, as it underestimates the vertebral rotation.^3^ In our cases (with Cobb’s angle greater than 50), a pre-operative CT scan aided us in identifying the defects in the inter-vertebral foramina, the distance, and direction from the skin to the inter-vertebral foramina. Instead of an inter-spinous approach, access to the lumbar epidural space was achieved through the inter-vertebral foramina on its convex side (to the right in patient 1 and the left in patient 2). A ‘click’ or a ‘pop’ like sensation followed by a loss of resistance to air identified the epidural space and catheters could be positioned. Injected local anaesthetic through the catheter provided adequate analgesia for right lower limb lengthening (required an additional dose) in patient 1 and patient 2 for proximal femoral nail.

With patients in a prone position, pain physicians employ the fluoroscopy or CT scan guided trans-foraminal technique to gain access to the anterior epidural space to deliver non-particulate steroid-LA mixtures resulting in improved pain scores.^4^ A pre-procedural ultrasound scan is recommended in the non-scoliotic spine^2^ before the procedure of lumbar epidural to recognise the relevant anatomical structures (ligamentum flavum and posterior surface of dura). But in limited patients with scoliosis, the neutral vertebra was identified with ultrasound to insert the epidural catheter. Further, the advances in ultrasound technology (needle tracking systems – Sonix GPS) would be of theoretical importance in the current scenario. The authors confess their inability to perform and analyse sonoanatomy of the scoliotic spine and relied on another radiological modality. A CT scan depicts the intervertebral foramen, the boundaries of which are the intervertebral disc anteriorly, the superior and inferior articular processes and the facet joint posteriorly, the roof and floor are formed by the pedicles of the respective levels. Several ligaments encroach the intervertebral foramen (corpora-transverse, extra-foraminal ligaments, and ligamentum flavum). The contents are the spinal nerve roots (L4 nerve root exits at L4-5 intervertebral disc level) covered by perineurium, an extension of the dura-mater, traverse while surrounded by arteries, veins, and epidural fat.^5^ Radicular arteries supply the spinal nerve and nerve roots within the intervertebral foramina and occupy the superior portion of the inter-vertebral foramen. The lower spinal cord is supplied by the artery of Adamkiewicz which originates on left, between T9 and L5 (23%), and is located in the superior portion of the inter-vertebral foramen. Accordingly, the needle tip is targeted in the posterior and lateral area of the defect, while the arterial arcade is in the superior portion of the intervertebral foramen.

In degenerative lumbar scoliosis, CT scan has revealed wedging of the inter-vertebral discs, translation, and rotation of the vertebral bodies and subluxation of the superior articular process. In the sagittal plane, cadaveric magnetic resonance images have revealed the cross-sectional foraminal area varies from 40 to 160 mm^2^, while an MRI study reported that the cross-sectional foraminal area was larger on the convex than the concave side in degenerative lumbar scoliosis.^6-8^ Using CT images, a forward and lateral flexion increased the cross-sectional foraminal area on the side opposite the flexion.^9^ Radio-anatomical studies support clinical findings that the lumbar radiculopathy exists more at the level of L5-S1^9^, which was avoided. Magnetic resonance imaging (MRI) of scoliosis (Cobb’s angle> 66 degrees) revealed horizontal width of epidural space between 3 to 5 cm on the convex side, and the dura sac pushed towards the concave side. Based on MRI, a modified paramedian approach in thoracic scoliosis has been described for thoracic epidural analgesia. The epidural catheter was inserted at a distance of 4 cm and positioned in the thoracic epidural space. Only with Cobb’s angle of 124 degrees, the epidural catheter had migrated into the intra-pleural space.^10^ Consequently, it would be prudent to approach the neuraxial space through the larger intervertebral foraminal defect on its convex side above the level of L5-S1.

The optimal distance of multi-orifice epidural catheters is between 3 and 5 cm.^11^ A CT scan study identified epidural catheter tip in the posterior epidural space, intervertebral foramina, and the paravertebral space after being inserted through the inter-spinous area in non-scoliotic patients.^12^ However, the dynamics of catheter tip positioning would be different in the scoliotic spine. On insertion at the level of convexity with the dura sac pushed towards the concave side the catheter migration will plausibly be prevented and would be retained on the convex side until a distance of 3 to 5 cm.

In severe lumbar scoliosis, when deemed necessary, a CT scan image obtained before the lumbar epidural procedure would be beneficial. In consultation with a radiologist, the level of the intervertebral foraminal defect (Figure 1B) through which would be the possible needle entry (Figure 1G), the direction and the angle with the sagittal plane in the axial view, and the final target point of the needle tip can be explored, analysed and established.

In our cases, the intervertebral foraminal defects were identified at the level of L3-4 on the left and L2-3 on the right of spinal columns which offered a ‘gateway’ to the neuraxial space. Under the guidance of radiologist markings for the insertion, the direction and the final target for the epidural needle were achieved.

Though the entire procedure seems to be infallible, it can stumble upon impediments. Counselling patient’s relatives and surgeons for CT scan to understand the anatomy of neuraxial space is a huge task. Consultation with a radiologist is mandatory for obvious reasons. Executing the procedure based on CT scan information is the final challenge. Further, targeting the posterior and lateral areas of the defect in the intervertebral foramina is challenging. Needle to nerve contact can cause severe paraesthesia, abnormal vascular structures in the foramen could be perforated and catheters could migrate in the anterior epidural space. Thus, it would require an experienced anaesthesiologist to perform these highly skilled blocks.

Though it would be justified to approach the neuraxial space with severe scoliosis through the inter-vertebral foramen on the convex side, alternatives like peripheral nerve blocks should be a part of the plan. However, surgeon’s denial in patient 1 and patient’s denial in the second case deferred the implementation of peripheral nerve blocks. Though an appropriate algorithm has been proposed for patients with mild-and-moderate scoliosis, it leaves us clueless considering the neuraxial approach in severe scoliosis. It is recommended that, if ultrasound of neuraxial space results in poor visibility, fluoroscopy be implemented. We doubt the role of fluoroscopy in the sitting position during neuraxial techniques on an operating theatre table. However, the approach to neuraxial space in the prone position is implemented by pain physicians, which would be wearisome in an orthopaedic patient. Apart from a well-organised plan with a preoperative CT scan identification of inter-vertebral foramen, needle trajectory, angle, and the final target in the inter-vertebral foramen, the use of stimulating catheter in the epidural space as an internal endpoint would be of added benefit in regional anaesthesiologist armamentarium.

## Conclusion

To conclude, an inter-vertebral foraminal approach to neuraxial space in patients with moderate-to-severe scoliosis appears justified if peripheral nerve blocks are a contraindication or refused. However, we emphasise that these should be employed by a skilled anaesthesiologist. To further maximise safety, perhaps a stimulating needle and catheter can be implemented. We advocate including both, the CT scan and the stimulating catheter in the existing algorithm.^2^
